# Risk Factors for Suicide Attempts Among Older Adults with Suicidal Ideation: A Cross-Sectional Study

**DOI:** 10.1093/geroni/igaf122.3056

**Published:** 2025-12-31

**Authors:** Gwang Suk Kim, Jae Jun Lee, Min Kyung Park, Layoung Kim, Sooyoung Kwon, Eun Ju Park, Hayejin Yang, Seungbum Yang

**Affiliations:** Yonsei University College of Nursing, Seoul, Republic of Korea; Yonsei University College of Nursing, Seoul, Republic of Korea; Yonsei University College of Nursing, Seoul, Republic of Korea; The University of Suwon, Hwaseong, Republic of Korea; Graduate School of Yonsei University, Seoul, Republic of Korea; Yonsei University College of Nursing, Seoul, Republic of Korea; Graduate School of Yonsei University, Seoul, Republic of Korea; Graduate School of Yonsei University, Seoul, Republic of Korea

## Abstract

Suicidal ideation is a significant public health issue among older adults; however, not all individuals with suicidal thoughts attempt suicide. Identifying the factors that distinguish people who attempt suicide from those who do not is essential for targeted prevention efforts. The aim of this study was to identify risk factors associated with suicide attempts among older adults with suicidal ideation. This cross-sectional study analyzed data from the 2021–2022 Korean Community Health Survey, a community-based nationwide survey. The study included adults aged 65 years and older expressing suicidal ideation within the past year. Independent variables included demographic, socioeconomic, lifestyle, and health-related factors; the dependent variable was suicide-attempt history within one year. Multiple logistic regression analysis was conducted to identify significant risk factors for suicide attempts. Among 14,626 older adults with suicidal ideation, 352 (2.4%) attempted suicide. Demographic and socioeconomic factors associated with higher suicide-attempt risk included younger age, women, financial difficulty (public welfare recipients), and living alone. Lifestyle and health-related factors, such as smoking, shorter sleep duration, depressive symptoms, higher perceived stress, and lower quality of life, were also significant predictors. These findings underscored the need for suicide-prevention efforts among older adults that prioritize mental-health support, financial and social assistance, sleep intervention, and smoking cessation. Identifying high-risk individuals through routine screening and providing tailored interventions may help prevent suicide attempts in this vulnerable population.

